# Functional Expression of Choline Transporters in the Blood–Brain Barrier

**DOI:** 10.3390/nu11102265

**Published:** 2019-09-20

**Authors:** Masato Inazu

**Affiliations:** 1 Institute of Medical Science, Tokyo Medical University, Tokyo 160-8402, Japan; inazu@tokyo-med.ac.jp; Tel.: +81-3-3351-6141; 2 Department of Molecular Preventive Medicine, Tokyo Medical University, Tokyo 160-8402, Japan

**Keywords:** choline, acetylcholine, transporter, blood–brain barrier, brain microvascular endothelial cells, central nervous system

## Abstract

Cholinergic neurons in the central nervous system play a vital role in higher brain functions, such as learning and memory. Choline is essential for the synthesis of the neurotransmitter acetylcholine by cholinergic neurons. The synthesis and metabolism of acetylcholine are important mechanisms for regulating neuronal activity. Choline is a positively charged quaternary ammonium compound that requires transporters to pass through the plasma membrane. Currently, there are three groups of choline transporters with different characteristics, such as affinity for choline, tissue distribution, and sodium dependence. They include (I) polyspecific organic cation transporters (OCT1-3: SLC22A1-3) with a low affinity for choline, (II) high-affinity choline transporter 1 (CHT1: SLC5A7), and (III) choline transporter-like proteins (CTL1-5: SLC44A1-5). Brain microvascular endothelial cells, which comprise part of the blood–brain barrier, take up extracellular choline via intermediate-affinity choline transporter-like protein 1 (CTL1) and low-affinity CTL2 transporters. CTL2 is responsible for excreting a high concentration of choline taken up by the brain microvascular endothelial cells on the brain side of the blood–brain barrier. CTL2 is also highly expressed in mitochondria and may be involved in the oxidative pathway of choline metabolism. Therefore, CTL1- and CTL2-mediated choline transport to the brain through the blood–brain barrier plays an essential role in various functions of the central nervous system by acting as the rate-limiting step of cholinergic neuronal activity.

## 1. Transport at the Blood–Brain Barrier

**Figure 1:**
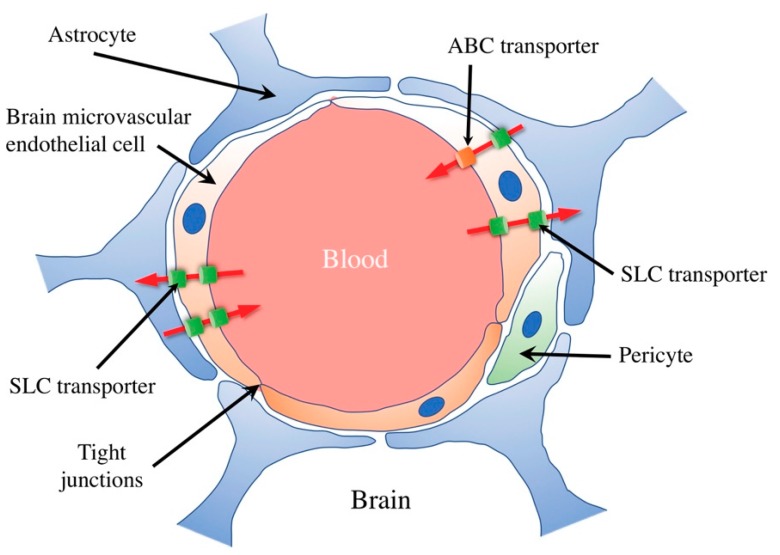
Schematic illustration of the blood–brain barrier and transporters. The blood–brain barrier is composed of brain microvascular endothelial cells, astrocytes, and pericytes. Diffusion between cells is limited by the mutual binding of brain microvascular endothelial cells by tight junctions. Many of the soluble carrier (SLC) transporters expressed in brain microvascular endothelial cells allow substances, such as nutrients (e.g., glucose, amino acids, peptides, and nucleotides), to selectively cross the blood–brain barrier. In addition, ATP binding cassette (ABC) transporters that are expressed in cerebral microvascular endothelial cells play a role in preventing the entry of toxic substances and drugs into the brain by releasing them into the blood.

The blood–brain barrier restricts the exchange of substances (e.g., nutrients, drugs, and poison) between the blood vessels of the brain and brain cells, and helps maintain the optimal environment for nerve function. Therefore, nutrients necessary for nerve activity in the brain (e.g., glucose and amino acids) are selectively transported, and unnecessary substances produced in the brain are excreted into the blood to maintain brain homeostasis. The blood–brain barrier is composed of brain microvascular endothelial cells, astrocytes, and pericytes. Diffusion between cells is limited by the adhesion of the brain microvascular endothelial cells to each other on the blood side [1] (Figure 1). As a result, substances must pass through the endothelial cells in order to be transferred from the circulating blood to the brain. Although highly water-soluble substances have difficulty crossing the blood–brain barrier, nutrients (e.g., glucose, amino acids, peptides, and nucleotides) selectively cross this barrier using various solute-carrier transporters (SLC transporters) that are expressed in the brain microvascular endothelial cells. In addition, ATP-binding cassette transporters (ABC transporters), which are also present in the brain microvascular endothelial cells, prevent the accumulation of a poison or drug in the brain by effluxing those that enter the brain back into the blood. Therefore, transporters present in the blood–brain barrier serve as gatekeepers that selectively restrict the transport of substances into the brain. This article reviews the functional expression of choline transporters and their role in the central nervous system.

## 2. Role of Choline in the Central Nervous System

**Figure 2:**
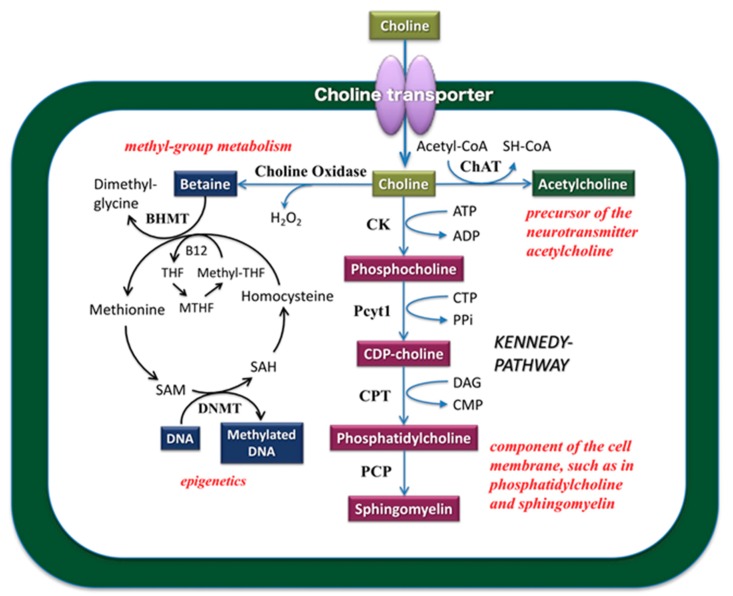
Choline metabolic pathway. Choline is an essential biological molecule for all cells and is required for the synthesis of phosphatidylcholine and sphingomyelin, which are the major components of the plasma membrane. New cell membrane synthesis requires the rate-limiting step of choline uptake, followed by phospholipid biosynthesis. Choline is also a precursor for the neurotransmitter acetylcholine and the methyl donor betaine, which are involved in several important biological functions. Betaine, an oxidized metabolite of choline, is a source of methyl groups for the production of S-adenosylmethionine (SAM), which serves as a substrate for DNA and histone methyltransferases, and is thus required for the establishment and maintenance of the epigenome. Epigenetic mechanisms play important roles in biology and human diseases. ADP, adenosine diphosphate; ATP, adenosine triphosphate; BHMT, betaine-homocysteine methyltransferase; VB12, vitamin B12; CDP, cytidine diphosphate; CK, choline kinase; CMP, cytidine monophosphate; CO, choline oxidase; CPT, choline phosphotransferase; DAG, diacylglycerol; CTP, cytidine triphosphate; methyl-THF, 5-methyltetrahydrofolate; MTHF, 5,10-methylene-tetrahydrofolate; PCP, phosphatidylcholine:ceramide choline phosphotransferase; Pcyt1, CTP:phosphocholine cytidyltransferase; PEMT, phosphatidylethanolamine N-methyltransferase; PLA, phospholipase A2; PPi, pyrophosphate; SAH, S-adenosylhomocysteine; SAM, S-adenosylmethionine; THF, tetrahydrofolate.

Choline is one of the biological factors which fulfill an important role for all cells, and after it is metabolized to the molecule necessary for the organism, it is mainly employed in three important physiological functions (Figure 2). The metabolism of choline is required for the synthesis of phospholipids (e.g., phosphatidylcholine and sphingomyelin), which are the main components of the cell membrane and S-adenosylmethionine, which is a methyl group donor. In the nervous system, choline is acetylated by choline acetyltransferase and used for the synthesis of the neurotransmitter acetylcholine, which is responsible for cholinergic nerve activity. The cholinergic nerves of the basal forebrain project into an extensive region of the cerebral cortex and are involved in various higher brain functions, such as sense and recognition, motion, memory, and learning. Choline deprivation in the central nervous system reduces acetylcholine release, memory, memory retention, and spatial cognition in the hippocampus [2,3,4]. In the brains of Alzheimer’s disease patients, the decrease in the number of nerve cells is remarkable in the Meynert basal nucleus, which is the origin nucleus of the cholinergic nerves of the cerebral cortex, and functional depression of the cholinergic nerves in the brain is an essential disease state [5]. In Alzheimer's disease, the core clinical manifestation is cognitive impairment accompanied by behavioral and psychiatric symptoms. In addition, it is hypothesized that choline is also involved in neurogenesis, including the self-replication of nerve stem cells and expansion of glial cells, through its role in phospholipid synthesis in the cell membrane. Choline metabolism is also linked to the synthesis of S-adenosylmethionine, which is involved in the methylation of DNA and histones, and serves as an epigenetics control mechanism in the central nervous system. Therefore, choline is an essential molecule involved in various functions in the central nervous system.

## 3. Characteristics of Choline Transporter Families

Choline uptake is dependent upon carrier-mediated transport because charged cations do not readily cross cell membranes by passive diffusion under physiological conditions. To date, the choline transport system is comprised of three transporter families. Each transporter possesses distinct properties, including choline affinity, tissue distribution, sodium dependence, sensitivity to the choline uptake inhibitor hemicholinium-3 (HC-3), and transport substrate [6] (Table 1).

The high-affinity choline transporter CHT1 (SLC5A7) is a marker for cholinergic neurons [7,8]. It is an Na^+^- and Cl^-^-dependent cotransporter that is highly sensitive to HC-3 and functions as part of the rate-limiting step of acetylcholine synthesis in cholinergic neurons. CHT1 knockout mice are characterized by hypoxia due to respiratory failure and consequently survive for only a few hours after birth [9]. This phenotype suggests impaired acetylcholine synthesis. Therefore, CHT1 is an essential protein for maintaining cholinergic neurotransmission by extracellular choline transport. CHT1 has also been identified in non-neuronal cholinergic cells (i.e., keratinocytes [10], lymphocytes [11,12,13], and ciliated cells of the tracheal epithelium [14]). Non-neuronal acetylcholine may function as a trophic molecule in paracrine and autocrine signaling.

Low-affinity choline transport systems are present in many tissues and supply choline primarily for the synthesis of phosphatidylcholine and other phospholipids, which are the major components of the plasma membrane [6,15,16]. The low-affinity and Na^+^-independent choline uptake systems consist of two transporter families: polyspecific organic cation transporters (OCT1-3/SLC22A1-3) and choline transporter-like proteins (CTL1-5/SLC44A1-5). 

The OCTs transport choline as a substrate. To date, three different OCTs have been identified and are primarily expressed in the kidney and liver. They function through voltage-dependent and Na^+^-independent uptake mechanisms [17,18,19]. These transporters recognize a large number of endogenous and exogenous organic cations as substrates and show considerable similarity in substrate specificity. Although OCT1 and OCT2 have low-affinity choline transporter activities, OCT3 does not recognize choline as a substrate [19,20]. 

There are currently five members of the CTL choline transporter family (CTL1-5) that are present in various human tissues [6,15,16,21,22]. CTL1 is an intermediate-affinity and Na^+^-independent choline transporter that can be inhibited by high concentrations of HC-3 or cationic drugs [23,24,25,26,27,28,29]. In addition, this choline transporter is a choline/H^+^ exchanger that uses a directional H^+^ gradient as the driving force, and its transport functions in cooperation with Na^+^/H^+^ exchangers [23,24,25,26,27,28,29]. The CTL1 protein is found in neuronal, glial, and endothelial cells of the rat and human central nervous system, suggesting that CTL1 transporter dysfunction may have important implications for the development and repair of the nervous system after injury and in neurodegenerative diseases [30]. CTL2 is highly expressed in the tongue, kidney, muscle, lung, and heart, but is found at low levels in the testes, intestine, and stomach [31]. In addition, CTL2 mRNA and protein are expressed in the human inner ear, and CTL2 is a target for antibody-induced hearing loss [32]. A CTL2 polymorphism constitutes human neutrophil alloantigen-3a [33]. Moreover, autoimmune disease is associated with a blockade of CTL2 function, which leads to an alteration in the hair cells in the inner ear and autoimmune hearing loss. Moderate CTL3 expression is present in the kidney, ileum, and colon, but there are no reports of its function. Very strong CTL4 expression is detected in the intestine, stomach, and kidney. It has been reported that human CTL4 protein functions as a thiamine pyrophosphate (TPP) transporter, but not a transporter of thiamine or choline [34,35]. Little information is available on the expression of CTL5, which was quite low in the brain and higher in the spinal cord, but at a lower level than CTL1 [31].

## 4. Functional Expression of Choline Transporters in Human Brain Microvascular Endothelial Cells

There have been many studies on the properties of choline transport in the blood–brain barrier. Choline transport in human brain microvascular endothelial cells occurs by an intermediate-affinity, Na^+^-independent uptake mechanism. Our lab identified and characterized the choline transporters expressed in human brain microvascular endothelial cells and the choline transport mechanisms in the blood–brain barrier [36]. CTL1 and CTL2 were the main choline transporters expressed in primary cultured human brain microvascular endothelial cells. Both transporters localized to the plasma membrane, and CTL2 was also present in mitochondria. Immunohistochemical staining of human cerebral cortex tissue sections confirmed that both of these transporters were expressed in brain microvascular endothelial cells, suggesting that choline transport in brain microvascular endothelial cells of the blood–brain barrier is regulated by both CTL1 and CTL2. 

**Figure 3:**
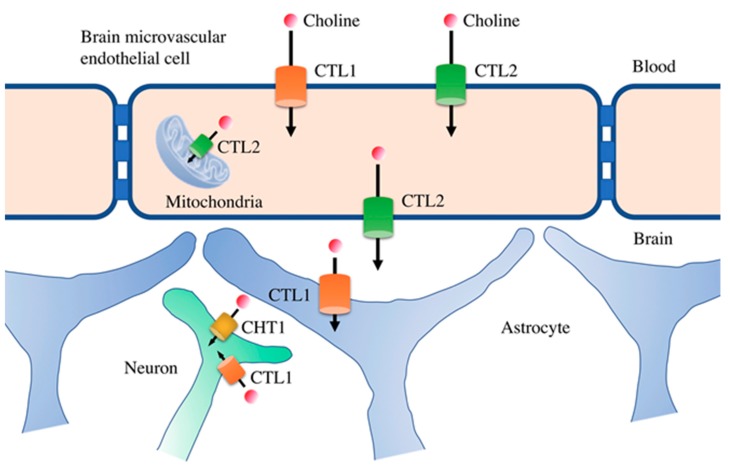
The localization of choline transporters in the blood–brain barrier. CTL1 and CTL2 are expressed in brain microvascular endothelial cells in the blood–brain barrier. Their localization on the luminal side of these cells suggests that they are responsible for the uptake of choline into the brain. CTL2 is also expressed on the apical side of brain microvascular endothelial cells, where choline is excreted into the brain. CTL1 is also expressed in astrocytes, one of the cell types that comprise the blood–brain barrier, and is thought to be linked to phospholipid synthesis. The high-affinity CHT1 and intermediate-affinity CTL1 are functionally expressed in neurons, where they may be involved in the synthesis of acetylcholine and phospholipids, respectively.

Functional analysis of choline uptake in human brain microvascular endothelial cells showed an Na^+^-independent and voltage-dependent uptake. In addition, two uptake mechanisms (intermediate-affinity, Km = 35 μM; low-affinity, Km = 54.1 μM) were identified. Specifically, intermediate-affinity choline transport is mediated by CTL1, and low-affinity choline transport is mediated by CTL2 (Figure 3). Because choline transporters with different affinities are expressed in the blood–brain barrier, the function of one transporter may be compensated for by the function of the other, thereby maintaining a constant choline concentration in the brain. The physiological blood concentration of choline ranges from 10 to 25 μM, suggesting that CTL1 is the major transporter for choline in the blood–brain barrier. CTL2 seems to play a role in the excretion of high concentrations of choline taken up by the brain microvascular endothelial cells through CTL1 on the brain side of the blood–brain barrier. CTL2 is also highly expressed in mitochondria, where it may be involved in the oxidative pathway of choline metabolism. Because choline oxidase, the enzyme that synthesizes betaine by choline oxidation, is localized in the inner membrane of mitochondria, it is thought that CTL2 is responsible for choline transport into mitochondria. Furthermore, S-adenosylmethionine, which is synthesized from betaine, is involved in the methylation of DNA and histones and is an important molecule for epigenetic control. Therefore, CTL2 may be involved in epigenetic control mechanisms, and the role of CTL2 in various epigenetic-mediated pathologies is of interest.

In the cholinergic nerve terminal, acetylcholine is synthesized from choline and acetyl-coenzyme A by choline acetyltransferase (ChAT) and then stored in synaptic vesicles by the vesicular acetylcholine transporter (VAChT). Acetylcholine is rapidly hydrolyzed to choline and acetic acid by acetylcholinesterase (AChE) or butyrylcholinesterase (BuChE) after synaptic release. It is known that acetylcholine is synthesized and released in cells other than neurons (e.g., vascular endothelial cells, keratinocytes, small cell lung carcinoma cells, immune cells, airway epithelial cells, the placenta, and astrocytes) to mediate various physiological functions (e.g., immune function and cell proliferation) [37,38,39]. We assessed whether choline uptake through both CTL1 and CTL2 in human brain microvascular endothelial cells is used for acetylcholine synthesis and found that ChAT, AChE, and VAChT mRNAs were not expressed, whereas BuChE was highly expressed, in these cells [36]. These results indicate that CTL1 and CTL2 are not involved in acetylcholine synthesis, and only the system for the degradation of endogenous acetylcholine by BuChE was present in human brain microvascular endothelial cells.

## 5. Potential of CTL1 and CTL2 as Drug Transporters

The blood–brain barrier possesses a variety of carrier-mediated transport systems to support and protect the brain function. Xenobiotic drugs recognized by influx transporters are expected to have a high permeability across the blood–brain barrier. In contrast, efflux transporters, including ATP-binding cassette transporters (e.g., P-glycoprotein) located at the luminal membrane of endothelial cells, function as clearance systems for metabolites and neurotoxic compounds produced in the brain. Many of the drugs known to be highly translocated into the brain are organic cationic substances, suggesting the existence of an unidentified organic cation transporter in the blood–brain barrier [40,41].

Choline exists as a cationic substance with a positive charge under physiological conditions. Choline transport is therefore expected to be competitively inhibited by cationic drugs. Indeed, choline uptake was inhibited in a concentration-dependent manner by the uptake of various cationic drugs (e.g., clonidine, desipramine, diphenhydramine, quinidine, quinine, and verapamil) in human brain microvascular endothelial cells [36]. These panel of compounds tested included central nervous system drugs (e.g., antidepressants) that might inhibit choline transport to the brain. These cationic drugs may also be recognized as transport substrates for choline transporters. Currently, the transporters of cationic drugs in the blood–brain barrier have not been elucidated. Therefore, an evaluation of CTL1 and CTL2 as drug transporters in human brain microvascular endothelial cells is warranted.

## 6. Role of Choline Transporters in the Central Nervous System

In the central nervous system, CTL1 is functionally expressed in neurons and astrocytes and thought to be primarily involved in the synthesis of phospholipids in the plasma membrane [23,42]. In contrast, CHT1 is expressed in cholinergic neurons and involved in acetylcholine synthesis [7]. Because the concentration of acetylcholine in the brain of CHT1 knockout mice did not differ from that of wild-type mice, CTL1 may transport the choline required for acetylcholine synthesis [9]. We previously found that choline transported by CTL1 was used for acetylcholine synthesis in neuroblastoma cells [24]. These findings suggest that CTL1 may compensate for choline transport when the function of the high-affinity CHT1, which normally transports the choline required for acetylcholine synthesis, is disrupted. For example, during cerebral ischemia, a decrease in the ATP content of nerve cells leads to a decrease in Na^+^/K^+^ ATPase activity, resulting in the intracellular accumulation of Na^+^ and a decrease in the Na^+^ concentration gradient. Under those circumstances, the Na^+^-dependent CHT1 function may be impaired. Alternatively, as an Na^+^-independent transporter, CTL1 can compensate for the function of CHT1. By clarifying the role of CTL1 in pathological conditions (e.g., cerebral ischemia), new therapeutic strategies may be found for these conditions. 

CTL1 expressed in astrocytes is presumably involved in astrocyte differentiation and proliferation; however, the mechanistic details are still unknown. A recent study demonstrated that a reduction of choline uptake by the hybrid neuroblastoma-glioma cell line NG108-15 significantly reduced cell growth [30]. Furthermore, CTL1 expression was increased in motor nuclei at the time of nerve regeneration following facial nerve transection [43]. These observations suggest that proliferation and repair may require an increased supply of choline. The expression of CTL1 in cholinergic neurons suggests that the transport activity of this transporter may play an important role in neurodegenerative diseases, such as Alzheimer’s disease. It is expected that its role in such diseases involving the cholinergic nerves will be clarified in the future.

## Figures and Tables

**Table 1:** Properties of choline transporters.

Protein Name	Km for Choline	Sodium-dependency	Sensitivity of HC-3 (Ki)	Tissue Distribution	Substrates
CHT1	0.5–3 µM	Yes	50–100 nM	Brain, spinal cord	Choline
CTL1	10–50 µM	No	10–100 µM	Multiple tissues	Choline, organic cation
CTL2	50–200 µM	Unknown	Unknown	Placenta, lung	Choline
CTL3	Unknown	Unknown	Unknown	Colon, pancreas	Unknown
CTL4	Unknown	Unknown	Unknown	Prostate, colon	Thiamine pyrophosphate
CTL5	Unknown	Unknown	Unknown	Multiple tissues	Unknown
OCT1	300–400 µM	No	>250 µM	Liver, kidney	Organic cation
OCT2	100–500 µM	No	>250 µM	Kidney, brain	Organic cation

CHT: high- affinity choline transporter, CTL: choline transporter-like protein, OTC: organic cation transporter, HC-3: hemicholinium-3.
